# The Quality of Online Orthopaedic Oncology Information

**DOI:** 10.5435/JAAOSGlobal-D-19-00181

**Published:** 2020-02-25

**Authors:** Ralph T. Zade, Jason P. Tartaglione, Ernest Chisena, Curtis T. Adams, Matthew R. DiCaprio

**Affiliations:** From the Orthopaedic Associates of Central Maryland Division, Catonsville, MD (Dr. Zade); the Ortho Rhode Island, Warwick, RI (Dr. Tartaglione); and the Division of Orthopedic Surgery, Albany Medical Center, Albany, NY (Dr. Chisena, Dr. Adams, and Dr. DiCaprio).

## Abstract

“Cancer” is one of the top three health-related Internet searches, yet research shows over 30% of patients are confused after searching for medical information. The quality and accuracy of Internet oncology literature varies widely and can affect patient perceptions or seeking of care.

**Purpose:**

This study hypothesizes that online patient resources for orthopaedic oncology are often inconsistent, inaccurate, or incomprehensible by the standard patient and examines the readability, quality, and accuracy of common orthopaedic oncology websites.

**Methods:**

Three common search terms were searched in three popular search engines. The first 25 nonsponsored websites were identified for each term; randomized to search term; and evaluated via a 25-question quality score, DISCERN treatment-based score, predetermined accuracy score, and Flesch-Kincaid reading level.

**Results:**

Forty-eight websites were included. Website quality, DISCERN score, accuracy score, and reading level were not statistically different based on search term. Quality and DISCERN scores were markedly higher from websites without commercial gain. Websites were consistently written above the recommended reading level.

**Discussion:**

Online orthopaedic oncology literature is frequently confusing and complicated. The orthopaedic surgeon should be aware that patients frequently access this information and should ensure that patients receive accurate primary source material relevant to their care.

According to the US Census Bureau, 83.8% of American households contain computers with Internet access.^1^ Seventy-two percent of those users access the Internet for health-related information.^2^ With a plethora of data available, the World Wide Web affords patients-increased availability of therapeutic information, social support, autonomy in the physician-patient relationship, and interaction with the healthcare system.^3-5^

While allowing easy access to information, Internet use for medical reference also has limitations. There is no standard method of review for online data and, thus patients may be subject to factually incorrect or outdated recommendations.^3,6,7,8,9^ Many websites also present industry-biased information that the average reader may interpret as legitimate and unbiased.^10,11^ Finally, search engines, the dominant source of health information,^2,12-14^ by necessity must filter the content and quality of information returned. Several studies have shown that results vary based on the user's search term.^11,15-17^

If patients do access websites with unbiased, peer-reviewed information, they may be unable to understand the data presented or may incorrectly interpret recommendations. Widely available medical and orthopaedic patient-oriented medical literature has been consistently shown to be more advanced than the sixth grade reading level recommended by the American Medical Association (AMA) and the National Institutes of Health for most patient comprehension.^15,16,17,18,19,20,21^ Up to 97% of patient education materials on the American Academy of Orthopaedic Surgeons website exceed a sixth grade readability level and 81% exceed an eighth grade level (national average).^21^ Because the AMA has cited health literacy as the strongest independent predictor of health status, these disparities can lead to poor patient outcomes.^22^

The quality and readability of websites pertaining to orthopaedic ailments such as distal radius fractures, shoulder instability, and scoliosis has been objectively evaluated.^11,15,17^ Although these conditions are relatively common, “cancer” is consistently one of the top three diseases searched on the Internet.^23,24^ In the primary care setting, bony lesions are often incidentally found on routine radiographs. Patient confusion over bony lesions or undiagnosed “tumors,” even if benign, can leave patients scouring the Internet for information. However, 30 percent of oncology patients are more confused after their initial search.^25^ This study hypothesizes that the quality, accuracy, and readability of Internet resources available to orthopaedic oncology patients through independent Internet searches is frequently inconsistent, confusing, and inadequately supported and that practitioners should remain the primary source of medical information for their patients.

## Methods

We selected two colloquial search terms to represent the presumed choices of a patient with a newly diagnosed radiographic bone lesion: “bone tumor” and “bone cancer.” We selected an additional, specific term representing the most frightening diagnosis a patient may come across: “sarcoma.” Each of the terms was independently entered into three commonly used search engines (Google, Bing, and Yahoo) on the same day. The first 25 results from each search (excluding sponsored advertisement links) were evaluated. Duplicates were eliminated, leaving 72 independent websites. Of these, 24 sites either lacked informational content or were unrelated to orthopaedic oncology and thus were excluded. Ultimately, 48 independent websites met the inclusion criteria and remained for assessment. A research assistant then randomized (from the initial order returned) and blinded (from the associated search term) all of the websites for evaluation to eliminate reviewer bias from perceived priority of website or responsiveness to search term.

The quality, accuracy, and readability of the websites were assessed using methods similar to previous investigations regarding shoulder instability, scoliosis, and distal radius fractures, with the addition of a validated questionnaire (DISCERN tool) to evaluate treatment recommendations.^11,15,17,26^ Thus, quality was evaluated using two criteria. A twenty-five-item grading sheet was developed based on the methods described in the aforementioned studies by the senior author (M.R.D.), a fellowship-trained orthopaedic oncologist. Questions were based on the elements of diagnosis, anatomy, treatment plan, and complications specific to bone sarcoma with attention to the format established on the American Academy of Orthopaedic Surgeons website for bone sarcoma in the upper extremity (Table 1).^27^ The second quality assessment criteria used the DISCERN questionnaire, a validated, 16-question instrument for assessment of written information on treatment choices for a health issue.^27^ Scores from the instrument range from 16 (low-quality treatment recommendations) to 80 (high-quality treatment recommendations). Three senior orthopaedic surgery residents then used the scoring sheet established by the senior author (M.R.D.) and DISCERN questionnaire to independently evaluate each website. Each resident had completed a formal orthopaedic oncology curriculum under the guidance of the senior author. All residents were not involved in randomization and were blinded to the search term that returned each website. The two quality scores from each reviewer were then aggregated and averaged to yield a mean quality score (maximum 25 points from the questionnaire and 80 points from the DISCERN instrument).

**Table 1:** Quality Scoring Criteria

Diagnosis
**•** Metastatic bone disease as most common in adults
**•** Discusses difference in benign versus malignant lesions
**•** Discusses different diagnoses based on age
**•** Discuses different tools for diagnosis (Radiograph, CT, MRI, bone scan, and biopsy)
Anatomy
**•** Discusses most common bones affected (distal femur, proximal tibia, and proximal humerus)
**•** Discusses regions of the bone affected (intramedullary, cortical, metaphyseal, and epiphyseal)
**•** Sarcomas can be aggressive and extend out of the bone
Treatment
**•** Treatment may involve chemotherapy
**•** Treatment may involve radiation therapy
**•** Treatment may involve surgery
**•** Treatment may be observation
**•** Surgery has two main purposes: tumor resection and reconstruction
**•** Sometimes treatment may be palliative instead of curative
**•** Patients may need stabilization if at risk for pathologic fracture
**•** Discusses surgical adjuvants (argon beam, phenol, and liquid nitrogen)
**•** Surgery may be initially delayed and patient restaged after neoadjuvant therapy
**•** Discusses limb salvage surgery versus amputation
**•** Notes treatment is multidisciplinary (medical oncologist, orthopaedic oncologist, radiation oncologist, musculoskeletal radiologist, musculoskeletal pathologist, physical medicine and rehabilitation, and pain specialist)
Complications
**•** Sarcoma can metastasize to other locations (commonly lungs/other bones)
**•** Lesions in weight-bearing bones may result in pathologic fractures
**•** Lesions may recur even if resected
**•** Patients must be monitored regularly after resection
**•** Surgery in children may result in growth disturbances/limb length inequality
**•** Wounds may become infected
**•** Surgery may require resection of structures such as muscles, vessels, and nerves that limit future function
Score _______/25

Accuracy of website information was graded on a scale of one to four based on previously established methods.^11,15,17,34^ A score of one was assigned if reviewers agreed with <25% of the website content, two if they agreed with 26% to 50% of content, three for agreement with 51% to 75%, and four for agreement with >75% of the website content. The scores of all three reviewers were then aggregated for each website to allow for interobserver variability and to create a composite accuracy score for each website with a maximum value of 12 possible points.

Readability was assessed using the Flesch-Kincaid (FK) method. The FK grade level indicates the maximum level of education a patient must have obtained to be able to comprehend the material.^28^ More complex reading materials correlate with higher scores. Website text was analyzed using the FK tool within Microsoft Word.^11,15,17,29,34^

Once the websites were scored, they were stratified in several different ways. In addition to grouping them according to the search term from which they were located, they were organized by the FK grade level. As previously stated, the AMA and the National Institutes of Health recommend a sixth grade reading level for patient education materials to be appropriate for the average (eighth grade) reading level of the US population.^18,19^ For the purposes of this study, the websites were then grouped according to those written at or below an eighth grade reading level and those written above an eighth grade reading level. Assuming that websites displaying advertisements generated profits from the products or companies they showcased, the websites were also stratified according to those with commercial gain, without commercial gain, and those sponsored by a medical treatment center. Treatment centers were chosen as a separate category because they typically generate profit from medical services, rather than merely providing medical information, a potential source of bias. Finally, the websites were categorized based on whether they were authored by a healthcare provider (physician, nurse, or physical therapist) or nonhealthcare provider (those websites that did not list authors were also included in this group).

Comparative statistics were used to analyze the content score, DISCERN rating, accuracy assessment, and FK level. For comparisons involving three categories (multiple search terms, sponsorship), one-way analysis of variance (assuming normally distributed data) with post hoc Tukey pairwise comparisons was used to determine significance. For comparisons involving two categories (authorship, reading level) independent sample *t*-tests were used to determine significance. Intraclass correlation coefficients were used to assess inter-relater reliability for content and DISCERN scores. The threshold for significance was *P* < 0.05 in all statistical methods. Statistical analysis was conducted using Excel 2011 (Microsoft) and Minitab 16 (Minitab) by a doctorate level statistician, Paul Feustel, PhD.

## Results

Of the 48 unique websites that were evaluated, 17 sites were found using the search term “bone tumor,” 14 sites using the term “bone cancer,” and 17 using the specific term “sarcoma” (Table 2). Healthcare providers (M.R.D. or R.N.) authored 20 sites, whereas nonhealthcare providers (or unknown authors) authored 28 sites. Twelve sites were associated with commercial bias, 18 sites were not associated with commercial bias, and 18 sites were sponsored by cancer treatment centers.

**Table 2:** Unique Websites Evaluated

• Cedars-Sinai.edu
• Bonetumor.org
• Nih.gov
• Mskcc.org
• Seattlecca.org
• Emedicinehealth.com
• Webmd.com
• Curesarcoma.org
• Cancer.gov
• Medical-dictionary.thefreedictionary.com
• Sarcoma.org
• Cancercenter.com
• Cancer.net
• Merckmanuals.com
• Mountsinai.org
• Orthoinfo.aaos.org
• Virginiamason.org
• Sarcomahelp.org
• Cancer.org
• Rightdiagnosis.com
• Nemsi.uchc.edu
• Radiologyassistant.nl
• Sarcomaalliance.org
• Mayoclinic.org
• Massgeneral.org
• Njms2.umdnj.edu
• Britannica.com
• Macmillan.org.uk
• Bonecancerresearch.org.uk
• Nytimes.com
• Hopkinsmedicine.org
• Healthline.com
• Cancer.about.com
• Childrenshospital.org
• Clevelandclinic.org
• Nhs.uk
• Sarcomaoncology.com
• Pubs.rsna.org
• Ucdmc.ucdavis.edu
• Wikipedia.org
• Cancerresearchuk.org
• Dana-farber.org
• Seattlechildrens.org
• Medicalnewstoday.com
• Orthopedics.about.com
• Medicinenet.com
• Bone.cancertreatment.net
• Patient.co.uk
• Mdanderson.org

As shown in Table 3, the average quality score for all websites was 12.4 ± 4.4 (maximum of 25 points). For “bone tumor,” the mean quality score was 13.1 ± 4.1 in comparison to “bone cancer,” which was 13.7 ± 3.7 and “sarcoma,” which was 10.7 ± 4.5. The average quality score for websites identified as written by a designated healthcare provider was 13.5 ± 4.5, which was not markedly different than those written by all other authors, which had a score of 11.7 ± 4.1 (*P* > 0.05). The average quality score for sites without commercial bias (14.0 ± 4.9) was markedly higher than the quality score of those with commercial bias (9.4 ± 4.4, *P* = 0.014) and higher without reaching statistical significance than those created by cancer treatment centers (12.9 ± 2.4, *P* > 0.05). The mean quality score for websites written above an eighth grade reading level (12.0 ± 3.8) was not significantly different from websites written at or below an eighth grade reading level (13.6 ± 5.5, *P* > 0.05).

**Table 3:** Summary of Quality Results

Indicator	Mean Quality (Maximum 25) ± SD
All websites	12.4 ± 4.4
Bone tumor	13.1 ± 4.1
Bone cancer	13.7 ± 3.7
Sarcoma	10.7 ± 4.5
Websites written by a physician	13.5 ± 4.5
Websites not written by a physician	11.7 ± 4.1
Websites without commercial bias	14.0 ± 4.9
Websites with commercial bias	9.4 ± 4.4
Websites from treatment centers	12.9 ± 2.4
Written above an eighth grade reading level	12.0 ± 3.8
Written at or below an eighth grade reading level	13.6 ± 5.5

As shown in Table 4, the average accuracy score for all websites was 10.3 ± 1.7 (maximum of 12 points). The mean accuracy score for “sarcoma” (9.5 ± 1.9) was lower than “bone tumor” (10.9 ± 1.2) and “bone cancer” (10.5 ± 1.5, *P* = 0.05); however, a 1-point accuracy difference may be clinically of limited relevance. There was no statistically significant difference in accuracy between results for “bone tumor” and “bone cancer.” Websites written by a healthcare provider had an average accuracy score of 10.8 ± 1.3 that was not significantly different from websites authored by nonphysicians, which had a mean accuracy score of 10.0 ± 1.8 (*P* > 0.05). The average accuracy score for sites with a commercial bias was 8.9 ± 1.6, which was significantly lower than sites without a commercial bias (11.1 ± 1.4, *P* < 0.001) and websites created by cancer treatment centers (10.4 ± 1.3, *P* = 0.026). There was no notable difference in accuracy between noncommercial and treatment center websites. The accuracy score for websites written above an eighth grade reading level was 10.3 ± 1.5, which was not significantly different than the accuracy score for websites written at or below an eighth grade reading level, which was 10.4 ± 2.0 (*P* > 0.05).

**Table 4:** Summary of Accuracy Results

Indicator	Mean Accuracy (Maximum 12) ± SD
All websites	10.3 ± 1.7
Bone tumor	10.9 ± 1.2
Bone cancer	10.5 ± 1.5
Sarcoma	9.5 ± 1.9
Websites written by a physician	10.8 ± 1.3
Websites not written by a physician	10.0 ± 1.8
Websites without commercial bias	11.1 ± 1.4
Websites with commercial bias	8.9 ± 1.6
Websites from treatment centers	10.4 ± 1.3
Written above an eighth grade reading level	10.3 ± 1.5
Written at or below an eighth grade reading level	10.4 ± 2.0

As demonstrated in Table 5, the average DISCERN treatment score for all websites was 46.1 ± 8.9 (maximum of 80 points). For “bone tumor,” the mean DISCERN score was 46.0 ± 7.8 as compared to “bone cancer,” which was 49.7 ± 8.6 and “sarcoma,” which was 43.2 ± 9.0; post hoc analysis did not demonstrate a significant difference between the three search terms (*P* > 0.05). The average DISCERN score for sites written by a physician was 48.5 ± 8.3, which was not significantly different than those written by all other authors, which had a score of 44.3 ± 8.9 (*P* > 0.05). The average DISCERN score for sites without commercial bias (52.0 ± 7.9) was significantly higher than sites with a commercial bias (39.8 ± 8.0, *P* < 0.001) and those created by cancer treatment centers (44.4 ± 6.4, *P* = 0.013). There was no notable difference between commercial and treatment center DISCERN scores. The mean DISCERN score for websites written above an eighth grade reading level was 45.0 ± 8.1 that was not significantly different from the mean DISCERN score of websites written at or below an eighth grade reading level, which was 49.3 ± 10.2 (*P* > 0.05).

**Table 5:** Summary of DISCERN Results

Indicator	Mean DISCERN (Maximum 80) ± SD
All websites	46.1 ± 8.9
Bone Tumor	46.0 ± 7.8
Bone cancer	49.7 ± 8.6
Sarcoma	43.2 ± 9.0
Websites written by a physician	48.5 ± 8.3
Websites not written by a physician	44.3 ± 8.9
Websites without commercial bias	52.0 ± 7.9
Websites with commercial bias	39.8 ± 8.0
Websites from treatment centers	44.4 ± 6.4
Written above an eighth grade reading level	45.0 ± 8.1
Written at or below an eighth grade reading level	49.3 ± 10.2

All websites were written above a Flesch-Kincaid (FK) sixth grade reading level and 75 percent (36/48) above an eighth grade reading level. As shown in Table 6, the overall FK score for all websites was 10.5 ± 1.4. The average FK score for websites written above an eight was 11.1 ± 0.9 as compared to a FK score of 8.5 ± 0.4 for websites written at or below an FK score of 8 (*P* < 0.001). The average FK score for “bone tumor” was 10.3 ± 1.6 that was not significantly different than either the average FK score for “bone cancer” (10.1 ± 1.2) or “sarcoma” (10.9 ± 1.3, *P* > 0.05). The mean FK score for sites written by a healthcare provider was 9.9 ± 1.5, which was significantly lower than those written by all other authors, which had a score of 10.9 ± 1.2 (*P* = 0.02). The average FK score for sites without a commercial bias was 10.5 ± 1.5, which was not significantly different than either websites with a commercial bias (10.1 ± 1.5) or websites created by cancer treatment centers (10.7 ± 1.2, *P* > 0.05).

**Table 6:** Summary of Flesch-Kincaid Results

Indicator	Mean FK Score ± SD
All websites	10.5 ± 1.4
Bone tumor	10.3 ± 1.6
Bone cancer	10.1 ± 1.2
Sarcoma	10.9 ± 1.3
Websites written by a physician	9.9 ± 1.5
Websites not written by a physician	10.9 ± 1.2
Websites without commercial bias	10.5 ± 1.5
Websites with commercial bias	10.1 ± 1.5
Websites from treatment centers	10.7 ± 1.2
Written above an eighth grade reading level	11.1 ± 0.9
Written at or below an eighth grade reading level	8.5 ± 0.4

## Discussion

Patients are increasingly turning to the Internet for health information or as an adjunct to physician-provided information.^30^ This is especially true in the setting of orthopaedic oncology because more than 60% of patients seek Internet guidance after new radiographic findings of a bone lesion.^24^ Unfortunately, they are often more confused after their search.^25^ Diaz et al^31^ found that 62% of patients prefer that doctors recommend specific websites regarding their medical diagnoses. Although previous studies have assessed the quality and accuracy of common orthopaedic websites,^32^ to our knowledge, this is the first to objectively assess the quality, accuracy, and readability of orthopaedic oncology websites using a validated website assessment tool and, more importantly, provide website recommendations for practitioners.

Contrary to previous studies evaluating Internet resources for distal radius fractures, shoulder instability, and scoliosis,^11,15,17^ our results demonstrated a markedly less accurate trend toward lower-quality (without reaching statistical significance) websites using the more specific search term “sarcoma” than “bone tumor” or “bone cancer.” There was no statistically significant difference in readability or DISCERN scores regarding search term. These findings may be attributed to several reasons. The order in which websites were returned was affected by the search term. “Sarcoma” initially returned many commercial, encyclopedia-type websites that defined without providing information on the specific medical term. The more colloquial terms “bone tumor” and “bone cancer,” however, initially returned more high-quality, patient-centered, noncommercial websites (47% and 43%, respectively, versus 24% for “sarcoma”). It is also possible that because of the emotionally charged nature of the diagnosis and large number of patients seeking information on oncology-related conditions, physicians may be adopting more household, patient-oriented terms for oncology websites compared with other orthopaedic topics. Similar findings were demonstrated in the analysis of websites found based on the search terms “hallux valgus” versus “bunion.”^33^ Finally, it is possible that the quality ratings of “sarcoma” websites would have been higher if a larger number of websites were analyzed.

With regard to website sponsorship, our findings indicate that websites without commercial affiliation provide markedly higher quality and more accurate information with better treatment recommendations. These results are similar to previous studies that have demonstrated higher quality information in websites without commercial bias.^11,15,17,34^ However, it is interesting that noncommercial websites also had markedly higher DISCERN scores for quality of treatment options (52.0 ± 1.9 versus 44.4 ± 1.5, *P* < 0.001) and a trend toward higher content quality (without reaching statistical significance) than treatment-center sponsored websites. Although treatment center websites are valuable and accurate, their purpose, in part, is to market their services toward patients. Thus it is plausible that their website content, especially regarding treatment recommendations, may be biased. This is concerning as patients may make inappropriate treatment decisions because they are misinformed to all available options. It is important for patients and clinicians to be aware of this potential bias because these websites may be the first resources accessed for information.

Despite the findings of variability in website quality and accuracy regarding sponsorship, we found no statistically significant difference in quality, accuracy, or DISCERN scores based on authorship. Interestingly, the websites written by healthcare providers were written at significantly lower Flesch-Kincaid scores than those websites written by nonphysicians (10.9 ± 1.3 versus 9.9 ± 1.5, *P* = 0.02). These findings are contrary to those of Garcia et al and Dy et al^11,16^ regarding shoulder instability and distal radius fractures, respectively, which demonstrated markedly higher website quality, accuracy, and reading level with websites authored by healthcare providers versus nonhealthcare providers. These results may be, in part, due to study design because websites without listed authorship, including many treatment-center websites, were contained in the “nonphysician” group. It is possible that if these websites were, in fact, written by physicians, the quality, accuracy, or reading level may have increased. It is also possible that, in support of the search term findings, orthopaedic oncologists and other healthcare providers are beginning to design websites that are more patient-oriented at lower reading levels.

Although physician-authored websites demonstrated markedly lower FK scores, it is concerning that the average website reading level in this study was still very high (10.5 ± 1.4). All websites were written above the AMA recommended sixth grade reading level and 75 percent (36 of 48) were written above an eighth grade reading level, which represents the US adult national average.^18,19^ In 2008, Badarudeen and Sabharwal^34^ demonstrated that from 2001 to 2008, the average FK level for pediatric information on the American Academy of Orthopaedic Surgeons website had increased from 8.7 to 8.9. Since that time, several studies examining reading materials in various other orthopaedic specialties have found even higher average reading levels consistent with our data.^11,15,17,34^ These findings suggest that more than half of the population is unable to comprehend the information presented on orthopaedic websites. Moreover, it seems that instead of improving the readability of orthopaedic websites, they are becoming more advanced. Although compared with previous studies^11,34^ our data demonstrated no statistically significant difference regarding quality or accuracy and reading level, patients are still unlikely to comprehend much of the orthopaedic oncology information presented on the Internet. In order for patient-oriented medical resources on the Internet to be beneficial to patients, an effort must be made to lower the reading level of patient websites, thereby avoiding confusion and potential psychoemotional harm because of misunderstood recommendations. The orthopaedic oncologist may play a notable role here in advising patients toward higher quality websites that they can understand.

Our study has several limitations. As an Internet-based study, our results are subject to change. The search results obtained for our data were created at a single time point and may be different from one moment to the next. In addition, we chose particular search terms to represent both colloquial and professional patient queries after the diagnosis of a new radiographic lesion. Different search terms may be used by patients (for example “osteosarcoma” instead of “sarcoma”); this could result in different websites populating an Internet search engine for a given patient. We chose the generic term “sarcoma” so as to not skew search results toward a specific oncologic diagnosis. After eliminating duplicate results, only 48 unique websites remained. It is possible that this is too few of a number to adequately assess the quality and accuracy of websites returned. However, previous studies have shown that Internet users are more likely to select websites listed on the first two pages returned from a search engine, supporting this method.^11^ Finally, based on the study design, websites were included under a particular search term if they were ranked higher in that search. Again, the reason for this design is that patients are more likely to select websites ranked higher in a search result. Several websites, however, were included under multiple search terms. Thus, it is possible that the quality and accuracy of the websites found under different search terms may have changed if all websites, regardless of order, were included for evaluation.

After finding a new radiographic lesion, patients may be fraught with fear and confusion and attempt to sort through the massive amount of data available on the Internet for answers. Unfortunately, based on our findings, it is clear that, similar to other orthopaedic diagnoses, many orthopaedic oncology websites are written at inappropriately high reading levels, biased by commercial enterprises, and lack quality and accuracy. Furthermore, unlike similar studies, our data demonstrate that many websites that may be sought first, including those sponsored by treatment centers, may not depict all available treatment options. Patients and clinicians must be aware of these inadequacies to avoid misinformation. Contrary to similar studies in other orthopaedic subspecialties, our results indicate that more colloquial search terms do not lead to lower quality or less accurate websites. Moreover, our study demonstrated that physician-authored sites were actually written at more understandable reading levels. Thus, it is possible that orthopaedic oncology websites are starting to evolve into more patient-centered entities. To that end, we have developed a list of five websites in our study that were ranked highest for quality, accuracy, and readability in an effort to provide some guidance for physicians (Table 7). Specifically, two of these (websites 1 and 2) demonstrate mean content scores greater than 20, DISCERN scores greater than 60, accuracy scores greater than 10, and readability at or below an eighth grade level.

**Table 7:** Recommended Patient Websites

http://orthoinfo.aaos.org (American Academy of Orthopaedic Surgeons)
http://www.macmillan.org.uk (Macmillan Cancer Support)
http://www.cancerresearchuk.org (Cancer Research UK)
http://www.cancer.org (American Cancer Society)
http://www.bonetumor.org (Bonetumor.org)

As previous research has demonstrated in other orthopaedic subspecialties, there is a distinct need for better developed Internet resources that are easily accessible via patient searches and that offer consistent, readily understandable material. Finally, this study emphasizes the role of orthopaedic surgeons in serving as the primary source of accurate and personalized medical information for patients and in providing intellectually and situationally appropriate medical information as part of overall patient care.
